# Identification of cellular retinoic acid binding protein 2 (CRABP2) as downstream target of nuclear factor I/X (NFIX): implications for skeletal dysplasia syndromes

**DOI:** 10.1093/jbmrpl/ziae060

**Published:** 2024-05-15

**Authors:** Kreepa G Kooblall, Mark Stevenson, Raphael Heilig, Michelle Stewart, Benjamin Wright, Helen Lockstone, David Buck, Roman Fischer, Sara Wells, Kate E Lines, Lydia Teboul, Raoul C Hennekam, Rajesh V Thakker

**Affiliations:** Academic Endocrine Unit, Radcliffe Department of Medicine, Oxford Centre for Diabetes, Endocrinology and Metabolism (OCDEM), University of Oxford, Churchill Hospital, Headington, Oxford OX3 7LJ, United Kingdom; Academic Endocrine Unit, Radcliffe Department of Medicine, Oxford Centre for Diabetes, Endocrinology and Metabolism (OCDEM), University of Oxford, Churchill Hospital, Headington, Oxford OX3 7LJ, United Kingdom; Target Discovery Unit, University of Oxford, Oxford OX3 7FZ, United Kingdom; MRC Harwell, Mary Lyon Centre, Harwell Science and Innovation Campus, Oxfordshire OX11 0RD, United Kingdom; Oxford Genomics Centre, The Wellcome Centre for Human Genetics, University of Oxford, Oxford OX3 7BN, United Kingdom; Oxford Genomics Centre, The Wellcome Centre for Human Genetics, University of Oxford, Oxford OX3 7BN, United Kingdom; Oxford Genomics Centre, The Wellcome Centre for Human Genetics, University of Oxford, Oxford OX3 7BN, United Kingdom; Target Discovery Unit, University of Oxford, Oxford OX3 7FZ, United Kingdom; MRC Harwell, Mary Lyon Centre, Harwell Science and Innovation Campus, Oxfordshire OX11 0RD, United Kingdom; Academic Endocrine Unit, Radcliffe Department of Medicine, Oxford Centre for Diabetes, Endocrinology and Metabolism (OCDEM), University of Oxford, Churchill Hospital, Headington, Oxford OX3 7LJ, United Kingdom; MRC Harwell, Mary Lyon Centre, Harwell Science and Innovation Campus, Oxfordshire OX11 0RD, United Kingdom; Department of Pediatrics, Amsterdam UMC, University of Amsterdam, Meibergdreef 9, 1105AZ Amsterdam, The Netherlands; Academic Endocrine Unit, Radcliffe Department of Medicine, Oxford Centre for Diabetes, Endocrinology and Metabolism (OCDEM), University of Oxford, Churchill Hospital, Headington, Oxford OX3 7LJ, United Kingdom

**Keywords:** MSS, downstream genes, RNA sequencing, proteomics, CRABP2, retinoic acid, VCAM1

## Abstract

Nuclear factor I/X (*NFIX*) mutations are associated with 2 skeletal dysplasias, Marshall-Smith (MSS) and Malan (MAL) syndromes. *NFIX* encodes a transcription factor that regulates expression of genes, including Bobby sox (*BBX*) and glial fibrillary acidic protein (*GFAP*) in neural progenitor cells and astrocytes, respectively. To elucidate the role of *NFIX* mutations in MSS, we studied their effects in fibroblast cell lines obtained from 5 MSS unrelated patients and 3 unaffected individuals. The 5 MSS *NFIX* frameshift mutations in exons 6-8 comprised 3 deletions (c.819-732_1079-948del, c.819-471_1079-687del, c.819-592_1079-808del), an insertion (c.1037_1038insT), and a duplication (c.1090dupG). Quantitative reverse transcription polymerase chain reaction (qRT-PCR) and western blot analyses using MSS and unrelated control fibroblasts and *in vitro* expression studies in monkey kidney fibroblast (COS-7) cells showed that frameshift mutations in *NFIX* exons 6-8 generated mutant transcripts that were not cleared by nonsense-mediated-decay mechanisms and encoded truncated NFIX proteins. Moreover, *BBX* or *GFAP* expression was unaffected in the majority of MSS fibroblasts. To identify novel NFIX downstream target genes, RNA sequencing and proteomics analyses were performed on mouse embryonic fibroblast (MEF) cells derived from control *Nfix^+/+^*, *Nfix^+/Del2^*, *Nfix^+/Del24^, Nfix^Del24/Del24^*, *Nfix^+/Del140^*, and *Nfix^Del140/Del140^* mice, compared with *Nfix^Del2/Del2^* mice which had developmental, skeletal, and neural abnormalities. This identified 191 transcripts and 815 proteins misregulated in *Nfix^Del2/Del2^* MEFs with ≥2-fold-change (*P* <0 .05). Validation studies using qRT-PCR and western blot analyses confirmed that 2 genes, cellular retinoic acid binding protein 2 (*Crabp2*) and vascular cell adhesion molecule 1 (*Vcam1*), were misregulated at the RNA and protein levels in *Nfix^Del2/Del2^* MEFs, and that *CRABP2* and *VCAM1* expressions were altered in 60%–100% of MSS fibroblast cells. Furthermore, *in vitro* luciferase reporter assays confirmed that NFIX directly regulates *CRABP2* promoter activity. Thus, these altered genes and pathways may represent possible targets for drugs as potential treatments and therapies for MSS.

## Introduction

Marshall-Smith syndrome (MSS; MIM ♯602 535) is a rare autosomal dominant disorder, characterized by growth retardation, short stature, distinctive facial features (comprising of a high forehead, proptosis, blue sclerae, anteverted nares, small and retracted mandible, gingival hypertrophy, and hypertrichosis), skeletal abnormalities, delayed motor and neural development, respiratory complications, and postnatal failure to thrive.^1^^,^^2^ MSS is caused by *de novo* heterozygous frameshift mutations clustered in exons 6 to 10 of the nuclear factor I/X (*NFIX*) gene (MIM ♯164 005).^3–5^

*NFIX*, located on chromosome 19p13.2, contains 11 exons and encodes 14 transcripts through differential splicing and the use of different transcription initiation sites, of which 11 are protein coding. NFIX isoform1, representing the canonical sequence, is a ubiquitously expressed 502 amino acid protein, which contains a conserved 194 amino acid N-terminal DNA binding and dimerization domain, and a C-terminal transactivation/repression domain of variable length, due to alternative splicing of exons 7 and 9. In mammals, the NFI gene family consists of 4 closely related genes (*NFIA*, *NFIB*, *NFIC*, and *NFIX*), which encode transcription factors that bind as homo- or heterodimers to the consensus palindromic sequence 5'-TTGGC(N5)GCCAA-3' present in the promoter regions of viral and cellular genes, to either activate or suppress transcription.^6^ The *NFIX* frameshift mutations reported in MSS patients disrupt the C-terminal transactivation or repression domain, and result in the production of aberrant transcripts that escape nonsense mediated mRNA decay (NMD), leading to dysfunctional mutant NFIX proteins that behave in a dominant negative manner.^3–5^ In contrast, entire gene deletions or heterozygous missense, nonsense, and frameshift *NFIX* mutations that predominantly affect exon 2, which encodes the N-terminal DNA binding and dimerization domain, typically lead to transcripts that are cleared by NMD. The resulting NFIX haploinsufficiency is associated with an overgrowth disorder called Malan (Sotos-like) syndrome (MAL; MIM ♯614 753), which is characterized by unusual facial phenotype, skeletal dysplasia, intellectual disability, and behavioral problems.^3^^,^^5^^,^^7^

The role of NFIX in MSS is currently poorly understood. NFIX has been reported to bind to the promoter region of the glial fibrillary acidic protein (*GFAP*) gene to activate gene transcription in astrocytes,^8^ while binding of NFIX to the Bobby Sox (*BBX*) enhancer element suppresses transcription in neural progenitor cells.^9^*GFAP* encodes intermediate filaments and is important for differentiation of cortical precursor cells into mature astrocytes during brain development^8^ and NFI family members have previously been reported to be important factors driving astrocyte differentiation during development of the central nervous system.^10^*BBX* encodes a transcription factor that binds to DNA to promote cell cycle progression from the G1 to S phase.^9^ In order to elucidate the role of *NFIX* mutations in MSS, we initially studied their effects on *GFAP* and *BBX* expression in fibroblast cell lines obtained from 5 MSS patients, and then utilized these fibroblasts and mouse embryonic fibroblasts (MEFs) derived from wild-type (*Nfix^+/+^*) mice, heterozygous (*Nfix^+/Del2^*, *Nfix^+/Del24^* and *Nfix^+/Del140^*) and homozygous (*Nfix^Del2/Del2^*, *Nfix^Del24/Del24^* and *Nfix^Del140/Del140^*) mutant mouse models^11^ in hypothesis-free approaches of RNA sequencing and proteomic analyses (Figure S1). Our approach led to the identification of 2 genes as novel downstream targets of NFIX, which may represent cellular pathways that could be used for targeted drug discovery as potential treatments for MSS patients.

## Materials and methods

### Study approval

Written informed consent was obtained from patients or their legal guardians, using protocols approved by the local and national ethics committees. All animal studies were approved by the Medical Research Council Harwell Institute Ethical Review Committee and were licensed under the Animal (Scientific Procedures) Act 1986, issued by the UK Government Home Office Department (PPL30/2433 and PPL30/3271).

### Fibroblast cell lines

Human fibroblast cells were obtained from 5 MSS patients, as previously reported.^3^^,^^4^ In addition, 3 unrelated control human fibroblast cell lines (CRL2072, CRL2106, CRL1475) were obtained from ATCC (LGC Standards, Middlesex, UK). Murine embryonic fibroblast (MEF) cells were prepared from embryonic day 13.5 *Nfix^+/+^*, *Nfix^+/Del2^*, *Nfix^Del2/Del2^*, *Nfix^+/Del24^, Nfix^Del24/Del24^*, *Nfix^+/Del140^* and *Nfix^Del140/Del140^* mice^11^ using standard protocols and immortalized by serial passaging. Cells were maintained in culture, as described in Supplemental Materials and Methods.

### Nucleic acid analyses, *in vitro* expression assays, RNA sequencing, proteomics, and *in silico* analyses

Extraction of DNA and RNA, DNA and RNA sequence analyses; generation of *NFIX* expression constructs, immunofluorescence, quantitative reverse transcription polymerase chain reaction (qRT-PCR), and western blot analyses; and dual luciferase reporter activity assays, chromatin immunoprecipitation (ChIP) followed by real time-PCR (ChIP-RT-PCR), RNA sequencing, proteomics, and *in silico* analyses, were performed as described in Supplemental Materials and Methods.

### Statistical analysis

Data were expressed as mean and SD or SEM. All analyses were performed using Prism (GraphPad), and a value of *P* <0 .05 was considered significant for all analyses, as described in Supplemental Materials and Methods.

## Results

### Confirmation of *NFIX* mutations in MSS patient fibroblasts and effects on *NFIX, BBX*, and *GFAP* expression

The fibroblast cell lines from the 5 MSS patients were confirmed to have heterozygous *NFIX* mutations in exons 6-8, and these comprised 3 deletions (c.819-732_1079-948del (Del1), c.819-471_1079-687del (Del2), c.819-592_1079-808del (Del3)), an insertion (c.1037_1038insT), and a duplication (c.1090dupG), resulting in truncated NFIX proteins^3^^,^^4^ (Figure S2). No coding *NFIX* mutations were detected in the fibroblasts from the 3 unrelated controls. The *NFIX* mutations did not affect *NFIX* mRNA levels in the 5 MSS fibroblast cell lines, which were not significantly different to those in the unrelated controls (Figure S3A), thereby confirming that the MSS mutant *NFIX* transcripts, as expected, are not cleared by NMD. However, NFIX protein expression could not be detected in any of the MSS or unrelated control fibroblasts by western blotting, indicating that NFIX protein levels were below the level of detection.

NFIX is reported to suppress *BBX* transcription but activate *GFAP* expression, in neural progenitor cells and astrocytes, respectively. However, there was no significant difference in *BBX* expression at either the mRNA (Figure S3B) or protein (Figures S3D–E) levels between fibroblasts from MSS patients or unrelated controls. In addition, there was also no significant difference in *GFAP* expression in 4 of the 5 MSS fibroblast cell lines, when compared with unrelated controls, while the MSS c.1090dupG was associated with reduced *GFAP* expression (-12 fold, *n* = 4, *P* <0 .05) at the mRNA (Figure S3C) and protein (-24 fold, *n* = 4, *P* <0 .01, Figure S3D–F) levels, when compared with the unrelated controls.

**Figure 1:**
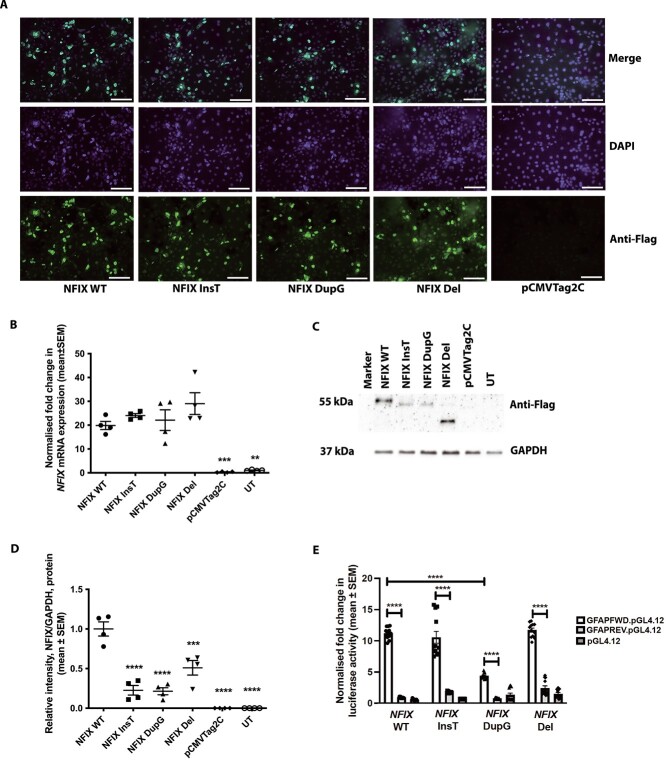
**Cellular localisation and functional studies of MSS mutants.** COS7 cells were transiently transfected with N-terminal Flag tagged WT (NFIX WT) or MSS-mutant (NFIX InsT, NFIX DupG, NFIX Del) *NFIX* cDNA constructs. pCMVTag2C was used as a negative control. (A) Cellular localisation of NFIX following immunofluorescence studies using an anti-Flag primary antibody and an Alexa Fluor 488 secondary antibody. NFIX proteins are predominantly located within the nucleus as determined by the overlap of the signal (teal colour) from the green fluorescent Flag tag on the NFIX protein and the blue DAPI stained nuclei. Scale bar represents 50 μm. (B) qRT-PCR analysis of *NFIX* expression normalised to *GAPDH* expression. (C) Western blots analysis using anti-Flag primary antibodies and HRP conjugated secondary antibody, and (D) quantified by densitometry analysis. Antibodies against GAPDH (37 kDa) were used as loading control. Untreated cells (UT) were used as an additional control. (E) *In vitro* dual luciferase reporter assays, in which the luciferase reporter gene is under the control of the *GFAP* promoter containing 3 NFIX binding sites, co-transfected with WT or mutant *NFIX* cDNA constructs. Data are represented as mean ± SEM, *n* = 4–12, ^*^^*^^*^^*^P <0 .0001, ^*^^*^^*^P <0 .001, ^*^^*^P <0 .01.

These effects of MSS-associated *NFIX* mutations were further investigated by *in vitro* expression assays in which N-terminal-Flag tagged *NFIX* wild-type (*NFIX* WT) and MSS-mutant (*NFIX* InsT, *NFIX* DupG, and *NFIX* Del comprising the loss of exons 6 and 7 that is representative of the deletions found in MSS fibroblast cell lines Del1, Del2 and Del3) cDNA constructs were transiently transfected into COS-7 cells. Immunofluorescence analysis showed that the *NFIX* mutations had no effect on the cellular localization of the NFIX protein with WT, InsT, DupG and Del NFIX proteins all predominantly located within the nucleus (Figure 1A). qRT-PCR analysis showed that there was no significant difference in *NFIX* mRNA expression between WT and the MSS-mutants (Figure 1B), thereby confirming the results obtained in the MSS fibroblasts. The combined use of NFIX overexpression and antibodies against the N-terminal-Flag tag allowed NFIX WT and mutant protein expression to be detected in the transiently transfected COS-7 cells by western blot analysis (Figure 1C and D). In each case the MSS-associated *NFIX* mutations resulted in the expression of truncated proteins of reduced molecular weight (<50 kDa) compared with WT NFIX (55 kDa) (Figure 1C). However, the levels of all mutant NFIX proteins were significantly lower than the level of WT NFIX (*n* = 4, *P* <0 .001, Figure 1C and D). These results further confirm that the MSS-associated *NFIX* mutations are transcribed and translated to produce mutant truncated proteins, and that mutations in the C-terminal part of the *NFIX* gene do not result in transcripts being cleared by NMD.

The observed reduction in *GFAP* expression associated with the *NFIX* c.1090dupG MSS mutation in the fibroblasts (Figure S3C, D, and F) was further assessed using reporter constructs comprising the luciferase reporter gene downstream of the *GFAP* promoter, which were transiently co-transfected with WT or MSS-mutant *NFIX* cDNA constructs into COS-7 cells. WT NFIX activated the *GFAP* promoter and caused a  ~12-fold increase (*n* = 4, *P* <0 .0001, Figure 1E) in luciferase activity in cells with the *GFAP* promoter cloned in the forward orientation compared with cells with the *GFAP* promoter cloned in the reverse orientation. In contrast, the NFIX DupG mutation caused a 2.5-fold reduction (*n* = 4, *P* <0 .0001) in luciferase activity compared with WT NFIX (Figure 1E), confirming a partial loss of function of NFIX transactivation activity at the *GFAP* locus, and consistent with results obtained in the MSS *NFIX* c.1090dupG patient fibroblasts. Luciferase reporter activity was unaffected by the MSS-associated *NFIX* InsT and Del mutations when compared with WT NFIX (Figure 1E).

### Identification of novel NFIX downstream target genes

The majority of the examined MSS-associated *NFIX* mutations did not act via the previously reported target genes, *BBX* or *GFAP*, and we therefore hypothesized that other downstream target genes may be involved in skeletal biology. To identify such novel NFIX downstream target genes that may be misregulated in MSS patients, we chose to initially undertake RNA sequencing and proteomics studies in MEFs because there is reduced genotypic variability in mice generated on the same genetic background compared with fibroblasts derived from unrelated MSS patients, thereby maximizing our chances of identifying statistically significantly altered pathways. We used MEFs derived from wild-type (WT; *Nfix^+/+^*) mice, and previously established *Nfix^+/Del2^*, *Nfix^+/Del24^*, *Nfix^+/Del140^*, *Nfix^Del2/Del2^*, *Nfix^Del24/Del24^* and *Nfix^Del140/Del140^* mutant mice^11^ that had deletions in exon 7 comprising: a 140 nucleotide deletion (Del140) that caused skipping of exon 7 due to alternative splicing of exon 6 to exon 8 and production of wild-type short *Nfix* isoforms that lack exon 7; an in-frame 24 nucleotide deletion (Del24) which caused loss of 8 amino acids; and a 2 nucleotide deletion (Del2) that caused a frame shift and a premature termination.^11^ Only the *Nfix^Del2/Del2^* mice showed a range of MSS phenotypes, while *Nfix^+/+^*, *Nfix^+/Del2^*, *Nfix^+/Del24^, Nfix^Del24/Del24^*, *Nfix^+/Del140^* and *Nfix^Del140/Del140^* mice were phenotypically normal.^11^ Expression of *Bbx* was found to differ by <2-fold between WT and mutant MEFs at the RNA level (Table S1), consistent with results obtained in the MSS human fibroblasts, but was absent at the protein level, while *Gfap* expression was below the level of detection in the WT and mutant MEFs.

RNA sequencing analysis identified 16 206 transcripts that were altered in the *Nfix^Del2/Del2^* MEFs compared with the mean of *Nfix^+/+^*, *Nfix^+/Del2^*, *Nfix^+/Del24^, Nfix^Del24/Del24^*, *Nfix^+/Del140^* and *Nfix^Del140/Del140^* MEFs, of which 191 transcripts had ≥2-fold-change (*P* <0 .05, Figure S4A). In parallel, proteomic analysis identified 4261 proteins that were altered in the *Nfix^Del2/Del2^* MEFs compared with the mean of *Nfix^+/+^*, *Nfix^+/Del2^*, *Nfix^+/Del24^, Nfix^Del24/Del24^*, *Nfix^+/Del140^* and *Nfix^Del140/Del140^* MEFs, of which 815 proteins had ≥2-fold-change (*P* <0 .05, Figure S4B). Comparison of the 191 RNA transcripts and 815 proteins that were altered ≥2-fold (*P* <0 .05) in the *Nfix^Del2/Del2^* MEFs compared with the other MEFs revealed 5 genes that were present in both datasets (Table S2). One gene, cellular retinoic acid binding protein 2 (*Crabp2*), was upregulated at both the RNA (2.59 fold, Table S2) and protein (2.83 fold, Table S2) levels, while 4 genes, vascular cell adhesion molecule 1 (*Vcam1*), potassium channel tetramerization domain containing 12 (*Kctd12*), isopentenyl-diphosphate delta isomerase 1 (*Idi1*), and eukaryotic translation initiation factor 2 subunit 3 structural gene Y-linked (*Eif2s3y*), were downregulated at both the RNA (-2.23 fold, -3.53 fold, -5.90 fold, and -7305.15 fold, respectively, Table S2) and protein (-4.89 fold, -4.77 fold, -2.14 fold, and -5.28 fold, respectively, Table S2) levels. Since there is no human ortholog for the mouse specific *Eif2s3y* gene, it was not considered for further analysis in relation to MSS. Thus, although there is 99% protein identity between human NFIX and the mouse homolog, one limitation of our approach is the exclusion in the identification of human specific genes.

### Validation of altered MEF gene expression in MSS human fibroblasts

**Figure 2:**
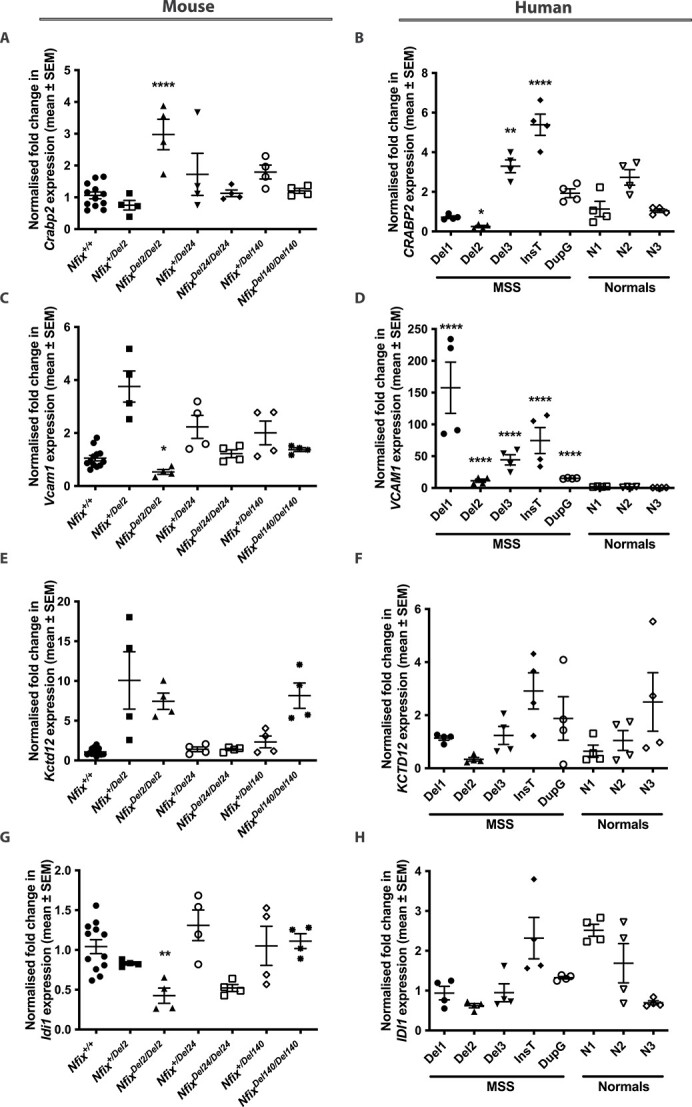
**QRT-PCR analysis of genes identified with altered expression in MEFs and human fibroblasts.** QRT-PCR analysis in: *Nfix^+/+^*, *Nfix^+/Del2^*, *Nfix^Del2/Del2^*, *Nfix^+/Del24^, Nfix^Del24/Del24^*, *Nfix^+/Del140^* and *Nfix^Del140/Del140^* MEFs; and 5 MSS (Del1, Del2, Del3, InsT and DupG) and 3 unrelated control (N1, N2 and N3) fibroblast cell lines, of (A) *Crabp2*, (B) *CRABP2,* (C) *Vcam1*, (D) *VCAM1*, (E) *Kctd12*, (F) *KCTD12*, (G) *Idi1* and (H) *IDI1* expression, with *Gapdh* or *GAPDH* and *Canx* or *CANX* used as the housekeeping genes against which candidate gene expression was normalised. Data are represented as mean ± SEM, *n* = 4–12, ^*^P <0 .05, ^*^^*^P <0 .01, ^*^^*^^*^^*^P <0 .0001.

**Figure 3:**
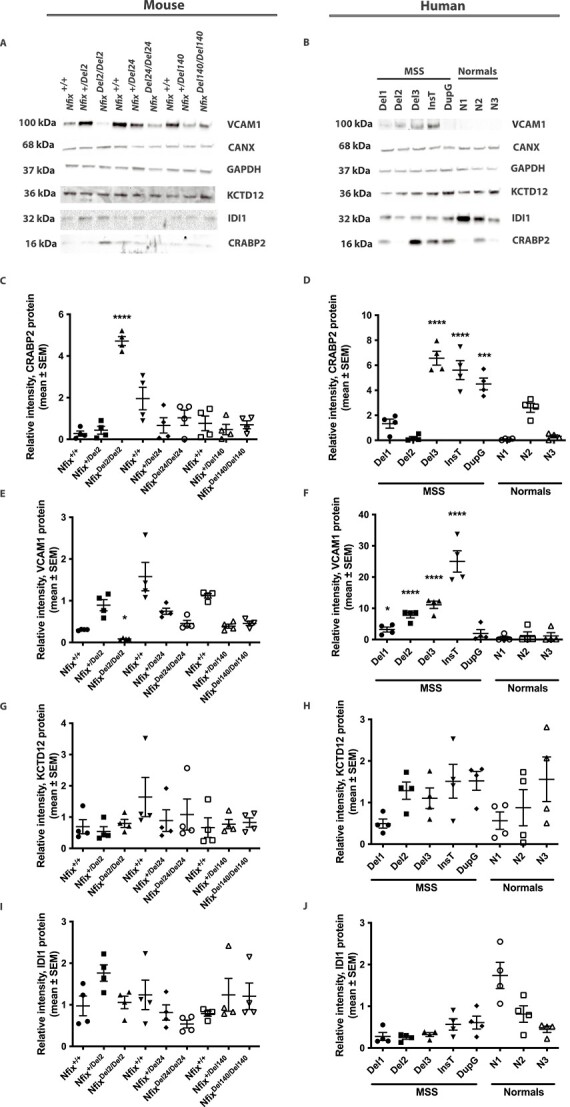
**Western blot and densitometry analysis of genes identified with altered expression in MEFs and human fibroblasts.** Western blot analysis in: (A) *Nfix^+/+^*, *Nfix^+/Del2^*, *Nfix^Del2/Del2^*, *Nfix^+/Del24^, Nfix^Del24/Del24^*, *Nfix^+/Del140^* and *Nfix^Del140/Del140^* MEFs; and (B) 5 MSS (Del1, Del2, Del3, InsT and DupG) and 3 unrelated control (N1, N2, and N3) fibroblast cell lines. Quantified expression, using densitometry analysis, of (C-D) CRABP2, (E-F) VCAM1, (G-H) KCTD12 and (I-J) IDI1 expression. GAPDH and CANX were used as loading controls. Data are represented as mean ± SEM, *n* = 4, ^*^P <0 .05, ^*^^*^P <0 .01, ^*^^*^^*^P <0 .001, ^*^^*^^*^^*^P <0 .0001.

To validate the changes observed in the expression of the *Crabp2*, *Vcam1*, *Kctd12* and *Idi1* genes revealed by the RNA and proteomic analyses in the MEFs, their expression in the MEFs and human fibroblasts was assessed by qRT-PCR (Figure 2A–H) and western blot (Figure 3A–J) analyses, respectively. *Crabp2* expression was confirmed by qRT-PCR analysis to be significantly increased in the *Nfix^Del2/Del2^* MEFs (2.4-fold, *P* <0 .0001), compared with the mean expression in the *Nfix^+/+^*, *Nfix^+/Del2^*, *Nfix^+/Del24^, Nfix^Del24/Del24^*, *Nfix^+/Del140^* and *Nfix^Del140/Del140^* MEFs (Figure 2A). Western blot analysis also showed significantly increased CRABP2 expression in *Nfix^Del2/Del2^* MEFs (6-fold, *P* <0 .0001, Figure 3A and C). Furthermore, *CRABP2* expression was significantly increased in the MSS human fibroblasts that had the c.819-592_1079-808del, Del3 (2-fold (*P* <0 .01) at the RNA level and 6.6-fold (*P* <0.0001) at the protein level), and c.1037_1038insT (3.3-fold (*P* <0 .0001) at the RNA level and 5.6-fold (*P* <0 .0001) at the protein level), when compared with the mean of the unrelated control human fibroblasts (Figures 2B and 3B and D). CRABP2 expression was also increased in the MSS human fibroblasts that had the c.1090dupG (4.5-fold change, *P* <0 .001), compared with unrelated controls, although this was only significant at the protein level (Figures 2B and 3B and D), possibly due to the high degree of variability in expression between the 3 unrelated control human fibroblasts. Surprisingly, *CRABP2* expression in the MSS human fibroblasts with the c.819-471_1079-687del (Del2) was reduced, compared with the unrelated control fibroblasts, although this was only significant at the RNA (0.2-fold change, *P* <0 .05, Figure 2B), and not the protein level (Figure 3B and D). These findings suggest that defects in NFIX generally lead to increased CRABP2 expression.

*Vcam1* expression was confirmed by qRT-PCR analysis to be significantly decreased in the *Nfix^Del2/Del2^* MEFs (0.3-fold, *P* <0 .05), compared with the mean expression in the *Nfix^+/+^*, *Nfix^+/Del2^*, *Nfix^+/Del24^, Nfix^Del24/Del24^*, *Nfix^+/Del140^* and *Nfix^Del140/Del140^* MEFs (Figure 2C). Western blot analysis also showed a significantly decreased VCAM1 expression in *Nfix^Del2/Del2^* MEFs (0.1-fold, *P* <0 .05, Figure 3A and 3E). However, qRT-PCR and western blot analyses of the MSS human fibroblasts showed that VCAM1 expression, in the presence of mutant NFIX proteins, was significantly increased at the RNA level by 109-fold, 8-fold, 31-fold, 52-fold, and 10-fold (all *P* <0 .0001) in the MSS human fibroblasts with the c.819-732_1079-948del (Del1), c.819-471_1079-687del (Del2), c.819-592_1079-808del (Del3), c.1037_1038insT, and c.1090dupG, respectively; and the protein level by 3-fold (*P* <0 .05), 8-fold (*P* <0 .0001), 11-fold (*P* <0 .0001), and 25-fold (*P* <0 .0001) in the MSS human fibroblasts with the c.819-732_1079-948del (Del1), c.819-471_1079-687del (Del2), c.819-592_1079-808del (Del3), and c.1037_1038insT, respectively (Figures 2D and 3B and F).

*Kctd12* expression, assessed by qRT-PCR and western blot analyses, in the MEFs did not reveal a significant difference between *Nfix^Del2/Del2^* MEFs and all the other MEFs (Figures 2E and 3A and G), thereby not supporting the RNA sequencing and proteomic data, and differences in *KCTD12* expression were also not detected in the human MSS and unrelated control fibroblasts (Figures 2F and 3B and H).

*Idi1* mRNA, but not IDI protein, expression was significantly decreased in the *Nfix^Del2/Del2^* MEFs (0.4-fold, *P* <0 .01), compared with the mean expression in the *Nfix^+/+^*, *Nfix^+/Del2^*, *Nfix^+/Del24^, Nfix^Del24/Del24^*, *Nfix^+/Del140^* and *Nfix^Del140/Del140^* MEFs (Figures 2G and 3A and I), and differences in *IDI1* expression were also not observed between unrelated controls and MSS human fibroblasts (Figures 2H and 3B and J). Thus, these findings do not generally support the earlier RNA sequencing and proteomic data.

Overall, expression of the *CRABP2* (*Crabp2*) and *VCAM1* (*Vcam1*) genes was found to be consistently altered in the *Nfix^Del2/Del2^* MEFs compared with all other MEFs, and in the majority of MSS human fibroblast cells, and we therefore focused further investigations on the promoters of these 2 genes.

### Effects of NFIX on *CRABP2* and *VCAM1* expression *in vitro*

**Figure 4:**
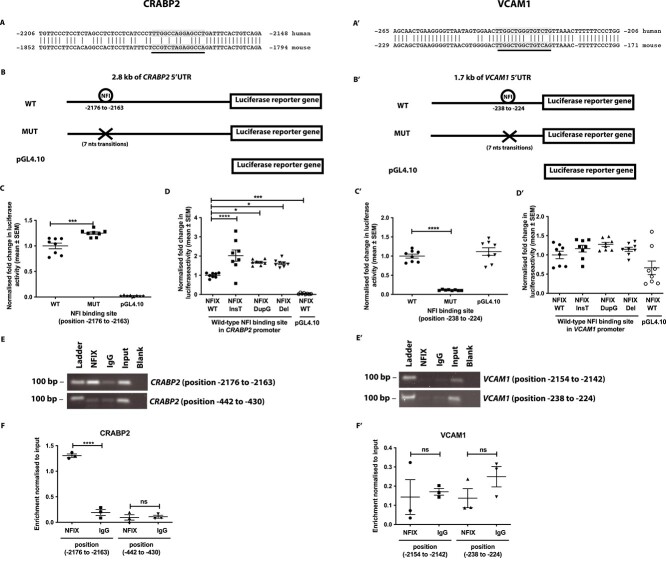
**Effects of NFIX on activities of *CRABP2* and *VCAM1* promoters.** Locations of a putative NFI binding sites (underlined) in the human and mouse genomes at positions: (A) −2176 to −2163 and −1822 to −1809 upstream of the ATG start site of the *CRABP2* and *Crabp2* genes, respectively; and (A’) −238 to −224 and −202 to −188 upstream of the ATG start site of the *VCAM1* and *Vcam1* genes, respectively. Luciferase reporter constructs were generated by cloning: (B) 2.8 kb of the *CRABP2* promoter, containing an NFI binding site located at position −2176 to −2163 upstream of the ATG start site; and (B’) 1.7 kb of the *VCAM1* promoter, containing an NFI binding site located at position −238 to −224 upstream of the ATG start site, upstream of the firefly luciferase reporter gene. *In vitro* dual luciferase reporter assays in COS-7 cells, in which the luciferase reporter gene is under the transcriptional control of the (C) *CRABP2* and (C’) *VCAM1* promoter, containing the wild-type (WT) or mutated (MUT; generated via the transition of 7 nucleotides) NFI binding site. A fourth construct lacking the entire promoter region (pGL4.10) was included as a control. *In vitro* dual luciferase reporter assays in COS-7 cells, in which the luciferase reporter gene is under the transcriptional control of the WT (D) *CRABP2* and (D’) *VCAM1* promoter, co-transfected with WT or MSS-associated mutant (NFIX InsT, NFIX DupG, NFIX Del) *NFIX* cDNA constructs. Chromatin immunoprecipitation using an anti-NFIX antibody or a non-specific IgG antibody compared to total input, coupled with real-time PCR (RT-PCR) was used to determine binding of NFIX to the NFI motifs identified in the *CRABP2* and *VCAM1* promoters. Two primers sets were designed to encompass either the identified: (E) −2176 to −2163 NFI motif or an unrelated −442 to −430 genomic site upstream of the ATG start site of *CRABP2* as an additional control; or (E’) −238 to −224 NFI motif or an unrelated −2154 to −2142 genomic site upstream of the ATG start site of *VCAM1* as an additional control. Enrichment of NFIX occupancy at the (F) *CRABP2* and (F’) *VCAM1* promoter relative to the IgG control was quantified as a percentage of total input. Data are represented as mean ± SEM, *n* = 3–8, ^*^P <0 .05, ^*^^*^^*^P <0 .001, ^*^^*^^*^^*^P <0 .0001.

*In silico* analysis of the 5’ untranslated regions of the *CRABP2* and *VCAM1* genes identified putative nuclear factor I (NFI) binding sites (palindromic sequence 5'-TTGGC(N5)GCCAA-3'), to which NFIX has been reported to bind.^12^ These comprised one potential NFI binding site in the human and mouse genomes at positions −2176 to −2163 and −1822 to −1809 upstream of the ATG start site of the *CRABP2* and *Crabp2* genes, respectively, which were conserved (Figure 4A); and another at positions −238 to −224 and −202 to −188 upstream of the ATG start site of the *VCAM1* and *Vcam1* genes, respectively, which were conserved (Figure 4A’).

**Figure 5:**
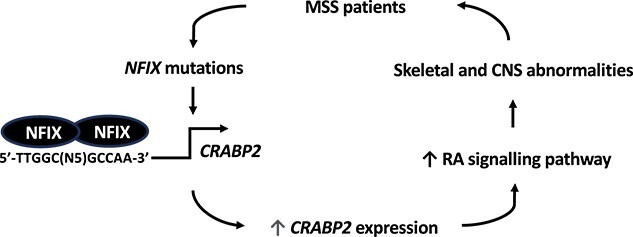
**Hypothesized role of NFIX on *CRABP2* expression in MSS patients.** *NFIX* mutations in MSS patients result in the production of dysfunctional NFIX proteins that directly bind to NFI binding sites (5'-TTGGC(N5)GCCAA-3') in the *CRABP2* promoter. Since mutant NFIX proteins have a reduced ability to suppress *CRABP2* promoter activity, this results in increased *CRABP2* expression, which in turn activates the RA pathway. Misregulation of *CRABP2* expression contributes to the skeletal and neurological phenotypes observed in MSS patients.

To investigate the effects of these identified putative NFI binding sites on promoter activity, luciferase reporter constructs under the transcriptional control of either the wild-type or mutant *CRABP2* (Figure 4B) or *VCAM1* (Figure 4B’) promoters, consisting of wild-type (WT) or mutated (MUT) NFI binding sites, respectively, were transfected into COS-7 cells. Mutation of the NFI binding site in the *CRABP2* promoter resulted in a 1.3-fold (*P* <0 .001) increase in luciferase expression compared with the wild-type *CRABP2* promoter (Figure 4C), while mutation of the NFI binding site in the *VCAM1* promoter resulted in 0.1-fold (*P* <0 .001) decrease in luciferase expression compared with the wild-type *VCAM1* promoter (Figure 4C’). Moreover, transient co-transfection of N-terminal-FLAG tagged wild-type (WT) or MSS-mutant *NFIX* cDNA constructs (InsT, DupG or Del) with these luciferase reporter constructs under the transcriptional control of wild-type *CRABP2* or *VCAM1* promoter in COS-7 cells revealed all the NFIX mutants to significantly increase luciferase reporter activity driven from the *CRABP2* promoter (2.0-fold (*P* <0 .0001), 1.7-fold (*P* <0 .05), and 1.6-fold (*P* <0 .05), respectively), but not from the *VCAM1* promoter, when compared with wild-type NFIX (Figure 4D and D’). Furthermore, chromatin immunoprecipitation (ChIP) followed by real time polymerase chain reaction (ChIP-RT-PCR) using DNA samples from the 3 unrelated control fibroblast cell lines revealed significant enrichment of NFIX binding to the -2176 to −2163 genomic site upstream of the ATG start site of *CRABP2* (*P* <0 .0001, Figure 4E and F), but not of the −238 to −224 genomic site upstream of the ATG start site of *VCAM1*, when compared with the non-specific IgG antibody (Figure 4E’ and F’). Thus, our studies confirm that NFIX can directly modulate the *CRABP2* promoter by suppressing its activity, and that NFIX may indirectly affect *VCAM1* expression, potentially via the retinoic acid (RA) pathway (Figure 5).

## Discussion

Our studies provide further insights of the roles of *NFIX* mutations in causing MSS. Thus, the fibroblast and *in vitro* expression studies confirmed that frameshift mutations in *NFIX* exons 6-8 generate transcripts that escape NMD and encode truncated NFIX proteins. Moreover, the majority of the studied MSS-associated *NFIX* mutations did not act via the known downstream targets, *BBX* or *GFAP* genes (Figure 1 and Figure S3). However, our RNA sequencing and proteomics studies identified *CRABP2* and *VCAM1* as new NFIX downstream target genes that were misregulated in 60%–100% of the MSS human fibroblasts and in *Nfix^Del2/Del2^* MEFs (Figures 2 and 3). Finally, *in vitro* expression and luciferase reporter assays confirmed that NFIX directly regulated the activity of the *CRABP2* promoter, and that NFIX may indirectly affect *VCAM1* expression (Figure 4), potentially via the RA pathway (Figure 5). These findings may help to provide some explanations for the occurrence of the range of skeletal and neuronal phenotypes in MSS patients as follows.

CRABP2, encoded by *CRABP2*, is a 138 amino acid (16 kDa) protein with strong binding affinity for RA and is a key component of the RA signaling pathway. RA is a metabolite and biologically active form of vitamin A, with complex and pleiotropic functions throughout life but particularly during embryogenesis where it regulates cell lineage and stem cell differentiation in developing tissue and organ systems, including the facial region, hindbrain, forebrain, eye and inner ear.^13^ CRABP2 regulates the expression of downstream genes in the RA pathway by binding to and transporting RA from the cytoplasm to the nucleus, where RA binds to its nuclear retinoic acid receptors (RARs) and peroxisomal proliferation-activated receptors, which form heterodimers with retinoid X receptors and associate with retinoic acid response elements in the promoter regions of RA-responsive genes to modulate transcription of target genes. RA degradation occurs via the action of cytochrome P450 (CYP) enzymes such as Cyp26A1 and Cyp26C1 that clear RA from the body.^13^ RA’s well-established effects during the development of numerous organs and tissues include skeletal growth, bone remodeling, and brain development.^13^ RA negatively influences osteoblast function, proliferation and differentiation as well as deposition of mineralized matrix.^13^ RA is required for normal bone development, acting as a morphogen during limb development^14^ as homozygous *Crabp2^−/−^* knockout mice were reported to be viable, normal and fertile with the exception for minor limb malformation, with variable penetrance depending on the genetic background.^15^ Since CRABP2 is important for RA function, its expression has been reported to be upregulated through the RA pathway in a permissive cellular context,^16^ and increased *CRABP2* expression is generally linked to the activation of the RA pathway.^13^ High retinol (vitamin A1) intake is associated with decreased bone mineral density and increased fracture risk in humans,^17–19^ and high dietary RA in mice has been shown to reduce bone mass by increasing osteoclastogenesis and decreasing osteoblastogenesis, without affecting bone mineralization.^20^ Moreover, short-term treatment of murine primary calvarial osteoblasts with RA induced an increase in *Crabp2* expression and other genes involved in retinol-dependent signaling and skeletal remodeling.^20^ In addition, homozygous *Crabp2^em1(IMPC)Mbp^* mice (C57BL/6N background) generated via CRISPR-Cas9-mediated deletion of exons 2–3 had heart abnormalities and decreased bone mineral content (International Mouse Phenotyping Consortium). Furthermore, the establishment of an RA gradient across the brain is required during development. In the forebrain, rostral-most tissues at the gastrula and neurula stages are believed to be devoid of RA signaling due to the action of Cyp26A1 and Cyp26C1.^13^ Administration of exogenous RA at such stages leads to drastic phenotypes with complete anencephaly or severe microcephaly due to inappropriate activation of RARs, which normally function as transcriptional repressors in anterior regions.^21^ Thus, altered *CRABP2* expression due to MSS-associated *NFIX* mutations could potentially disrupt RA signaling and lead to the skeletal abnormalities and increased fracture risk, as well as CNS anomalies that are observed in MSS patients.^22^

VCAM1, encoded by *VCAM1*, is a 739 amino acid (81 kDa) cell surface glycoprotein adhesion molecule that is a member of the Ig-superfamily of transmembrane proteins, and is expressed by cytokine-activated endothelial cells^23^ with its expression being upregulated in response to inflammatory cytokines, chemokines and growth factors.^24^ Increases in VCAM1 expression in macrophages, fibroblasts and endothelial cells have been reported in rheumatoid arthritis,^25^ while *Vcam1* expression in murine osteoblasts is important for cellular adhesion and activation of osteoblasts to binding partners in bones, which are required for maintaining the signals regulating the balance between bone formation and bone resorption.^26^ VCAM1 is also important for the regulation of hematopoietic stem cells self-renewal, proliferation, differentiation, trafficking, mobilization and function in the bone marrow,^27^^,^^28^ and conditional *Vcam1* deletion in mice is reported to impair lymphocyte migration to the bone marrow.^29^ However, Vcam-1-deficient mouse embryos are not viable and die due to severe defects in placental and heart development.^30^ VCAM1 is also reported to maintain the integrity of the mesenchymal and neural stem cell niches and to act as an environmental sensor to injury and disease.^31^^,^^32^ For example, mesenchymal stem cells that reside in the perivascular niche of many organs can proliferate after kidney, lung, liver or heart injury to generate myofibroblasts^32^ and are a major source of osteoblast-like cells during vascular calcification in a mouse model of chronic kidney disease.^33^ Finally, elevated VCAM1 levels have been reported in patients with extensive cerebral small vessel disease,^34^ dementia,^35^ Alzheimer’s disease,^36^ Parkinson’s disease^37^ and small vessel stroke.^38^ This suggests that increased VCAM1 expression in the endothelial cells of the CNS may have pathological actions, which can potentially exacerbate cognitive dysfunction. In contrast, in experimental neurodegenerative disease and age-related cognitive dysfunction models, treatment with dietary supplements of alpha lipoic acid resulted in downregulation of VCAM1, reduced inflammatory cell infiltration into the CNS and improved memory.^39^ Therefore, increased expression of *VCAM1* due to MSS-associated *NFIX* mutations may potentially contribute to CNS and bone abnormalities as well as other anomalies such as renal cysts, nephrocalcinosis, hydronephrosis, congestion of the liver and spleen, cardiomegaly, right ventricular hypertrophy and heart failure,^1^^,^^11^^,^^40^^,^^41^ which are reported in MSS patients.^22^

VCAM1 appears to be an indirect target of the RA pathway, possibly via the actions of intermediate transcription factors, non-classical associations of receptors with other proteins, or even more distant mechanisms.^16^ The *VCAM1* promoter has restricted activity due to the presence of strong negative regulatory elements (silencers) situated between -1.7 kb and -288 bp upstream of the transcription start site.^42–44^ In addition, the *VCAM1* promoter has 2 important tandem nuclear factor kappa-β (NF-κβ) elements (activators) that are required to overcome transcriptional inhibition, which are responsible for cytokine-dependent activation of the *VCAM1* promoter.^42–44^ Reported inducers of the *VCAM1* promoter include thrombin, tumor necrosis factor alpha (TNF-α) and RA.^42–44^ However, the responsiveness of the *VCAM1* promoter to RA appears to be cell-specific due to the variable distribution, number and combination of silencers and activators between cell types. For example, RA has been reported to activate p50/p65 induced *Vcam1* expression in mouse neuroepithelial cells, which are precursors for neurons and glia,^43^ while in human dermal microvascular epithelial cells RA has been shown to inhibit TNF-α induced *VCAM1* expression.^45^ Moreover, RA has been reported to significantly increase VCAM1 antigen expression in human neuroblastoma cells.^46^

Our results showed variability in the expression of the NFIX downstream target genes *CRABP2* and *VCAM1* (Figures 2 and 3), especially between mouse and human fibroblasts, which might not be indicative of changes in gene expression in other skeletal or non-skeletal cell types and *in vivo,* and this could be due to cell autonomous, monoallelic, and stochastic variation in NFIA, NFIB, NFIC and NFIX expression. Since the NFI family members are ubiquitously expressed in partially overlapping patterns and have the same conserved N-terminal DNA binding and dimerization domain, all 4 related genes may recognize the same consensus sequence present in the promoter region of downstream target genes, and may compensate for one another in case of mutation by changing their expression pattern and acquiring new regulatory capabilities in order to provide functional redundancy for the mutation.^10^^,^^11^^,^^22^ Moreover, MSS patients are heterozygous for *NFIX* mutations, and this contrasts with *Nfix^+/Del2^* mice which are normal, while developmental, skeletal and neural abnormalities are observed in *Nfix^Del2/Del2^* mice.^11^ Such differences between organisms are not uncommon and can be attributed to allelic variation, modifier genes, genetic variations, genetic background and environmental conditions in animal models *versus* in patients.^47–49^ An example of allelic variations is provided by the autosomal dominant disorder spondyloepimetaphyseal dysplasia, Missouri type (SEMD_MO_) which in humans is due to a heterozygous matrix metalloproteinase 13 (*MMP13*) missense F56S mutation, but in mice occurs only in homozygous *Mmp13^-/-^* mice deleted for exons 3, 4 and 5 that have defects in growth plate cartilage and delayed endochondral ossification, while heterozygous *Mmp13^+/–^* mice have normal growth plates and no skeletal abnormalities. The phenotypic differences between humans and mice in this case were demonstrated to be due to auto-catalytic MMP13 enzyme activity induced by the mutant which degraded the WT MMP13, thereby exerting a dominant effect and leading to a deficiency in MMP13 that was similar in *Mmp13^-/-^* mice, which developed skeletal abnormalities resembling those observed in patients SEMD_MO._^50^

In summary, we report the identification of *CRABP2* and *VCAM1* as NFIX downstream target genes that are misregulated in 60%-100% of the MSS human and mouse fibroblasts. Thus, NFIX may directly regulate the activity of the *CRABP2* promoter and *NFIX* mutations may alter *CRABP2* expression in MSS patients, and NFIX may indirectly affect *VCAM1* expression via the RA signaling pathway (Figure 5). Interestingly, CRABP2 expression has been reported to be upregulated through the RA pathway, while VCAM1 appears to be an indirect target of the RA pathway in a permissive cellular context.^16^ This suggests that dysfunctional NFIX proteins in MSS may directly increase *CRABP2* expression through a reduced ability to suppress *CRABP2* promoter activity which in turn results in the activation of the RA pathway, which may then indirectly misregulate *VCAM1* expression in a tissue-specific manner, and contribute to the pathology observed in MSS (Figure 5). This may indicate a possible misregulation of the RA pathway in MSS patients, and the possibility that drugs targeting the RA pathway might be of therapeutic benefit to MSS patients.

## Supplementary Material

Supplemental_Figures_and_Tables_rebuttal_clean_ziae060

Supplemental_Materials_and_Methods_ziae060

## Data Availability

The datasets generated and/or analyzed for this study can be obtained from the corresponding author on reasonable request. All data needed to evaluate the conclusion in the paper are presented in the article and in its online supplementary material.
