# Supplementary material for: Identification of cellular retinoic acid binding protein 2 (CRABP2) as downstream target of nuclear factor I/X (NFIX): implications for skeletal dysplasia syndromes
Source: JBMR Plus. 2024 May 15;8(7):ziae060. doi: 10.1093/jbmrpl/ziae060 (PMC11144382; doi:10.1093/jbmrpl/ziae060)
Supplement: Supplemental_Materials_and_Methods_ziae060 [file supplemental_materials_and_methods_ziae060.docx]

**Supplemental Materials and Methods**

**Fibroblast cell lines**

All human and murine fibroblasts were maintained in Dulbecco’s Modified Eagle medium: nutrient mixture F-12 (DMEM/F-12) (Life Technologies, Paisley, UK), supplemented with 100U/ml penicillin, 100μg/ml streptomycin and 10% heat-inactivated fetal calf serum (FCS). Monkey kidney fibroblast (COS-7) cells (CRL-1651) were obtained from ATCC (LGC Standards) and maintained in Roswell Park Memorial Institute (RPMI) 1640 medium (Life Technologies), supplemented with 10% heat-inactivated FCS and glutamine. All cells were incubated at 37°C, 5% CO_2_ and 95% humidity.

**Nucleic acid analysis**

Genomic DNA from fibroblast cells was isolated using DNA extraction buffer (10 mM NaCl, 20 mM Tris-HCl, pH 8.0, 1 mM EDTA, 10% SDS; MilliporeSigma, Cambridge, UK) and 0.3 mg/mL Proteinase K solution (Thermo Fisher Scientific, Manchester, UK) as previously described^(1)^. Genotyping of human fibroblasts was performed by polymerase chain reaction (PCR) amplification using *NFIX* specific primers (MilliporeSigma) as previously described,^(2);^ ^(3)^ followed by dideoxynucleotide Sanger DNA sequencing using the BigDye Terminator v3.1 Cycle Sequencing Kit (Life Technologies) and an ABI automated detection system (ABI 3730 Automated capillary sequencer, Applied Biosystems, Loughborough, UK). All *NFIX* exons and intron/exon boundaries were sequenced and compared with the human *NFIX* Ensembl reference sequence (ENSMUSG00000008441). A multiplex ligation-dependent probe amplification (MLPA) kit P300 (MRC Holland, Amsterdam, The Netherlands) was used to analyse variations in *NFIX* gene copy number, according to local protocols^(3)^.

**Reverse transcription polymerase chain reaction (RT-PCR)**

Total RNA was extracted from the fibroblasts using the RNeasy Mini Kit (Qiagen, Manchester, UK). RNA concentration and quality were determined using a NanoDrop ND-1000 Spectrophotometer (NanoDrop Technologies, Loughborough, UK), and RNA integrity determined by agarose gel electrophoresis. Up to 1µg of total RNA was converted to cDNA using the QuantiTect Reverse Transcription Kit (Qiagen). RT-PCR reaction was performed by PCR amplification using 5’-GGGGTCTGGAATGTGACG-3’ and 5’-CTCGACTGCTGGATGATGG-3’ primers (MilliporeSigma) in exons 4 and 8, respectively, as previously described^(3)^. The products were separated by agarose gel electrophoresis and visualised using a Bio-Rad Chemidoc XRS+ system UV transilluminator (Bio-Rad). The products were excised from the agarose gel, cleaned using the Qiaquik gel extraction kit (Qiagen) according to the manufacturer’s instructions, followed by dideoxynucleotide Sanger DNA sequencing using the BigDye Terminator v3.1 Cycle Sequencing Kit (Life Technologies) and an ABI 3730 automated capillary sequencer (Applied Biosystems). Sequences were compared with the human *NFIX* Ensembl reference sequence (ENSMUSG00000008441) to confirm the deletion of exons 6 and 7 mutations.

**Generation of *NFIX* expression constructs**

Wild-type human *NFIX* cDNA (I.M.A.G.E clone ID: 3491917) (SourceBioscience, Nottingham, UK) was cloned into the N-terminal-Flag pCMVTag2C expression vector (Promega, Southampton, UK). The complete deletion of exons 6 and 7 (c.819_1079del; *NFIX* Del), the insertion in exon 7 (c. 1037_1038insT; *NFIX* InsT) and the duplication in exon 8 (c.1090dupG; *NFIX* DupG) mutations were introduced by site-directed mutagenesis using the QuikChange Lightning Site-Directed Mutagenesis kit (Agilent, Cheshire, UK).

**Immunofluorescence**

COS-7 cells, seeded at 50-70% confluence, were transiently transfected with 0.5µg/well of wild-type or mutant *NFIX* cDNA expression constructs using Fugene transfection reagent (Promega). After 48h, transfected or mock transfected COS-7 cells were fixed on coverslips using ice cold 100% methanol for 10 min, permeabilised with ice cold 0.25% Triton X-100/ phosphate-buffered saline (PBS) for 10 min and blocked with 1% bovine serum albumin (BSA)/ PBS-Tween (PBST) for 30 min at room temperature. The cells were then incubated with 1:500 mouse anti-Flag primary antibody (AB-1257, Abcam, Cambridge, UK) followed by 1:1000 anti-mouse Alexa Fluor 488 secondary antibody (Invitrogen, Leicester, UK). The cells were mounted with ProLong Gold Antifade reagent (Invitrogen), visualised using a Leica model DM4000B microscope, and images captured using a Leica Microsystems DFC320 digital camera (Leica Microsystems, Milton Keynes, UK).

**Quantitative reverse transcription- PCR (qRT-PCR)**

Total RNA was extracted from human and murine fibroblast cells using the RNeasy Mini Kit (Qiagen). RNA concentration and quality were determined using a NanoDrop ND-1000 Spectrophotometer (NanoDrop Technologies), and RNA integrity determined by agarose gel electrophoresis. Up to 1µg of total RNA was converted to cDNA using the QuantiTect Reverse Transcription Kit (Qiagen). qRT-PCR was performed using human *NFIX*, *BBX*, *GFAP, CRABP2, VCAM1, KCTD12, IDI1*, *CANX* and *GAPDH* specific, and murine *Crabp2*, *Vcam1*, *Kctd12*, *Idi1, Canx* and *Gapdh* specific QuantiTect primers (Qiagen) and the QuantiTect SYBR Green PCR Kit (Qiagen), using a Rotor-Gene Q Cycler (Qiagen), and methods as previously described^(4)^. The relative expression of the target cDNA was normalised against the *GAPDH* and *CANX* or *Gapdh* and *Canx* cDNA controls using the Pfaffl method^(5)^.

**Western blot**

Total protein was extracted from human and murine fibroblast cells using ice-cold lysis buffer (150mM NaCl, 50mM Tris (pH 8.0), 1% Triton X-100 (v/v) and 1x Protease inhibitor (Roche, Hertfordshire, UK)). Protein concentration was determined using a Bradford assay, and samples were prepared with 4x Laemmli loading dye (BioRad, Hertfordshire, UK) and boiled at 95°C for 5 min prior to being resolved using precast 4-12.5% SDS-PAGE gel electrophoresis (BioRad). Protein samples were then transferred onto PVDF membrane (PerkinElmer, Cambridge, UK), blocked in 5% milk in PBS and incubated with appropriate primary antibodies comprising 1:2000 mouse anti-Flag (AB-1257, Abcam), 1:1000 rabbit anti-BBX (AB-94418, Abcam), 1:5000 rabbit anti-GFAP (AB-7260, Abcam), 1:1000 rabbit anti-CRABP2 (AB-211927, Abcam), 1:2000 rabbit anti-VCAM1 (AB-134047, Abcam), 1:100 rabbit anti-KCTD12 (AB-176201, Abcam), 1:1000 rabbit anti-IDI1 (AB-97448, Abcam), 1:5000 rabbit anti-CANX (AB-10286, Abcam) or 1:5000 rabbit anti-GAPDH (AB-9485, Abcam) in 5% milk/PBS-T. Membranes were washed and subsequently incubated with 1:2000 anti-mouse HRP-conjugated or 1:2500 anti-rabbit HRP-conjugated secondary antibodies (SC-2004, Santa Cruz Biotechnology, Dallas, USA) and visualised using ECL Western blotting substrate (BioRad) on a Chemidoc XRS+ system (BioRad) and densitometry analysis performed using Image J software, as previously described^(4)^.

**Dual luciferase reporter activity assays**

The human *GFAP* promoter, consisting of a 1.7 kb promoter region including the transcription start site and three NFIX binding sites, was cloned upstream of a promoter-less firefly luciferase gene (*luc*) in pGL4.12 (Promega), using methods as previously described^(4,6,7)^. In addition, a reporter firefly luciferase construct in which the *GFAP* promoter was cloned in the reverse orientation (GFAPREV.pGL4.12) and the pGL4.12 empty vector were included as additional controls. Moreover, the human *CRABP2* and *VCAM1* promoter, consisting of a 2.8 kb or 1.7 kb promoter region respectively, including the transcription start site and one potential NFI binding site, was cloned upstream of a promoter-less firefly luciferase gene (*luc*) in pGL4.10 (Promega), using methods as previously described^(4,6,7)^. The mutant reporter firefly luciferase constructs in which the NFI binding site was obliterated via 7 nucleotides transition, were generated by site-directed mutagenesis using the QuikChange Lightning Site-Directed Mutagenesis kit (Agilent) and the following primers:

VCAM1FWDMut: 5'-GAGCAACTGAAGGGGTTAATAGTGGAACCCAACTGGGTACTTGTTAAACTTTTTTCCCTGGCTCTGCCC-3'; VCAM1SDMREVMut: 5'-GGGCAGAGCCAGGGAAAAAAGTTTAACAAGTACCCAGTTGGGTTCCACTATTAACCCCTTCAGTTGCTC-3'; CRABP2FWDMut: 5'-GTTCCCTCCTCTAGCCTCTCCTCATCCCTCCAACCAGGAATTTGATTTCACTGTCAGAGCCTAATC-3'; CRABP2REVMut: 5'-GATTAGGCTCTGACAGTGAAATCAAATTCCTGGTTGGAGGGATGAGGAGAGGCTAGAGGAGGGAAC-3'. The pGL4.10 empty vector was included as a control. COS-7 cells, at 50-70% confluence, were transiently co-transfected with 0.5µg/well of a luciferase reporter construct together with 0.05µg/well of *Renilla* luciferase (pRL-TK) co-reporter vector as an internal control (Promega), and 0.5µg/well of either wild-type or mutant *NFIX* cDNA expression constructs, using Fugene transfection reagent (Promega), as previously reported^(4)^. After 48h, the cells were lysed and dual luciferase reporter activity determined using the Dual-Luciferase Reporter Assay System (Promega) and a Veritas Microplate Luminometer (Turner BioSystems, Southampton, UK), as previously described^(4)^. The ratio of the firefly luciferase to *Renilla* luciferase reporter activity was normalised against the activity in cells that were mock-transfected with the empty vectors.

**RNA sequencing analysis**

RNA sequencing analysis was used to investigate transcriptome differences in MEFs derived from the wild-type or mutant mice. Total RNA was isolated from *Nfix^+/+^*, *Nfix^+/Del2^*, *Nfix^Del2/Del2^*, *Nfix^+/Del24^,* *Nfix^Del24/Del24^*, *Nfix^+/Del140^* and *Nfix^Del140/Del140^* MEFs using the RNeasy Mini Kit (Qiagen). RNA concentration and quality were determined using a NanoDrop ND-1000 Spectrophotometer (NanoDrop Technologies), and RNA integrity was determined using agarose gel electrophoresis. RNA sequencing was performed using the Illumina HiSeq platform at the Oxford Genomics Centre (Wellcome Trust Centre for Human Genetics, University of Oxford), and the transcriptome was analysed using Ingenuity Pathway Analysis software (Qiagen).

**Proteomics analysis**

Proteomic analysis was used to identify differences in protein expression in MEFs derived from the wild-type and mutant mice. Proteins were extracted from *Nfix^+/+^*, *Nfix^+/Del2^*, *Nfix^Del2/Del2^*, *Nfix^+/Del24^,* *Nfix^Del24/Del24^*, *Nfix^+/Del140^* and *Nfix^Del140/Del140^* MEFs, using ice-cold lysis buffer (150mM NaCl, 50mM Tris (pH 8.0), 1% Triton X-100 (v/v) and 1x Protease inhibitor (Roche)), maintained in constant agitation at 4°C for 30 min, centrifuged for 10 min at 10,000 rpm and the supernatant collected. Protein concentration was determined using a Bradford assay. 100 ug protein was reduced and alkylated using with DTT and iodoacetamide, followed by chloroform/methanol precipitation and resuspension in TEAB for digestion with trypsin. Digested proteins were then labelled with TMT10 reagents according to manufacturer’s instructions, pooled and subjected to high-pH pre-fractionation into 10 fractions. Each fraction was then analysed by LC-MS/MS on an Orbitrap Fusion Lumos (Thermo Fisher Scientific) mass spectrometer connected to a Dionex Ultimate 3000 UPLC system. Resulting data was analysed with Proteome Discoverer software (V2.1) using a Mascot node (Matrix Science Ltd) and standard TMT processing parameters. The mass spectrometry proteomics data have been deposited to the ProteomeXchange Consortium via the PRIDE^(8)^ partner repository with the dataset identifier PXD_tbd.

***In silico* analysis**

MotifMap, the integrative genome-wide maps of regulatory elements,^(9,10)^ (<http://motifmap.ics.uci.edu/>) was examined for potential nuclear factor I (NFI) binding sites (5'-TTGGCNNNNNGCCAA-3’) in the 5’ untranslated region (UTR) of the *CRABP2* and *VCAM1* genes. NFI motifs were searched in the human and mouse genomes using the term ‘NF-1’ and limited within 10 kb of the promoter region. The database was last accessed on 5^th^ August 2021.

**Chromatin immunoprecipitation real time-PCR (ChIP-RT-PCR)**

Fibroblast cells were fixed in 1% formalin in culture media before being split into four aliquots and sonicated to produce DNA fragments between 200 and 1000 bp. Chromatin Immunoprecipitation (ChIP) was undertaken using the ChIP assay kit (Merck Millipore) with samples incubated overnight with 8 μg anti-NFIX (SAB1401263, Sigma) antibody. DNA from each ChIP was un-cross-linked, purified using phenol extraction, precipitated using ethanol and resuspended in nuclease-free water. For all experiments, IgG (AB-108-C, R&D Systems) and input (no antibody) controls were also included for analysis. Real time-PCR (RT-PCR) was performed using primer sets spanning the identified NFI binding sites as well as a negative control primer sets in regions devoid of transcription factor binding for the *CRABP2* and *VCAM1* promoters. Enrichment of antibody binding in the *CRABP2* and *VCAM1* promoters was calculated relative to input control.

**Statistical analysis**

Data are expressed as mean and standard deviations (SD) or standard errors of mean (SEM). Statistical analysis to compare two groups was performed using a two-tailed Student’s *t-*tests. When comparing more than two groups, statistical analysis was performed using ANOVA with Bonferroni correction for multiple testing applied^(4)^. All analyses were performed using Prism (GraphPad), and a value of p < 0.05 was considered significant for all analyses.

**References**

1. Lemos MC, Harding B, Shalet SM, Thakker RV. A novel MEN1 intronic mutation associated with multiple endocrine neoplasia type 1. Clinical Endocrinology. 2007;66(5):709-13.

2. Malan V, Rajan D, Thomas S, Shaw AC, Picard HLD, Layet V, et al. Distinct Effects of Allelic NFIX Mutations on Nonsense-Mediated mRNA Decay Engender Either a Sotos-like or a Marshall-Smith Syndrome. American Journal of Human Genetics. 2010;87(2):189-98.

3. Schanze D, Neubauer D, Cormier-Daire V, Delrue M-A, Dieux-Coeslier A, Hasegawa T, et al. Deletions in the 3 ' Part of the NFIX Gene Including a Recurrent Alu-Mediated Deletion of Exon 6 and 7 Account for Previously Unexplained Cases of Marshall-Smith Syndrome. Human Mutation. 2014;35(9):1092-100.

4. Newey PJ, Gorvin CM, Cleland SJ, Willberg CB, Bridge M, Azharuddin M, et al. Mutant Prolactin Receptor and Familial Hyperprolactinemia. New England Journal of Medicine. 2013;369(21):2012-20.

5. Pfaffl MW. A new mathematical model for relative quantification in real-time RT-PCR. Nucleic Acids Research. 2001;29(9).

6. Dixon C, Harvey TJ, Smith AG, Gronostajski RM, Bailey TL, Piper M. Nuclear Factor One X Regulates Bobby Sox During Development of the Mouse Forebrain. Cellular and Molecular Neurobiology. 2013;33(7):867-73.

7. Singh SK, Wilczynska KM, Grzybowski A, Yester J, Osrah B, Bryan L, et al. The Unique Transcriptional Activation Domain of Nuclear Factor-I-X3 Is Critical to Specifically Induce Marker Gene Expression in Astrocytes. Journal of Biological Chemistry. 2011;286(9):7315-26.

8. Perez-Riverol Y, Bai JW, Bandla C, García-Seisdedos D, Hewapathirana S, Kamatchinathan S, et al. The PRIDE database resources in 2022: a hub for mass spectrometry-based proteomics evidences. Nucleic Acids Research. 2022;50(D1):D543-D52.

9. Daily K, Patel VR, Rigor P, Xie XH, Baldi P. MotifMap: integrative genome-wide maps of regulatory motif sites for model species. Bmc Bioinformatics. 2011;12.

10. Xie XH, Rigor P, Baldi P. MotifMap: a human genome-wide map of candidate regulatory motif sites. Bioinformatics. 2009;25(2):167-74.
