# Supplementary material for: Identification of cellular retinoic acid binding protein 2 (CRABP2) as downstream target of nuclear factor I/X (NFIX): implications for skeletal dysplasia syndromes
Source: JBMR Plus. 2024 May 15;8(7):ziae060. doi: 10.1093/jbmrpl/ziae060 (PMC11144382; doi:10.1093/jbmrpl/ziae060)
Supplement: Supplemental_Figures_and_Tables_rebuttal_clean_ziae060 [file supplemental_figures_and_tables_rebuttal_clean_ziae060.docx]

**Supplemental Figure S1. Flow diagram showing the study.** RNA sequencing and proteomic analyses led to the identification of 191 transcripts and 815 proteins that had altered expression with ≥ 2-fold-change, respectively, in the *Nfix^Del2/Del2^* MEFs compared to the mean of *Nfix^+/+^*, *Nfix^+/Del2^*, *Nfix^+/Del24^*, *Nfix^+/Del140^*, *Nfix^Del24/Del24^* and *Nfix^Del140/Del140^* MEFs. Five genes were selected based on their misregulation at both the RNA and protein levels for further validation studies in MSS fibroblast cells.

**Supplemental Figure S2. Sanger sequence analysis of *NFIX* using genomic DNA and cDNA from MSS and unrelated control fibroblasts.** Sanger DNA sequencing analyses of human fibroblast cell lines using *NFIX* specific primers spanning the 11 coding regions of the *NFIX* gene (exons 1 to 11) and intron/exon boundaries were used to sequence the entire *NFIX* coding region to confirm the previously reported mutations in the 5 MSS fibroblasts^(3,4)^. DNA sequence chromatograms of genomic DNA are indicated relative to the translation start site showing: (A) wild-type genomic *NFIX* exon 7 in an unrelated control individual (N1) and mutant genomic *NFIX* exon 7 in an affected MSS individual with an insertion (c.1037_1038insT (InsT); p.Thr347Hisfs*76) in exon 7; and (B) wild-type genomic *NFIX* exon 8 in an unrelated control individual (N1) and mutant genomic *NFIX* exon 8 in an affected MSS individual with a duplication (c.1090dupG (DupG); p.Ala364Glyfs*59) in exon 8. In order to confirm the large intronic deletions in the three remaining MSS patients, *NFIX* cDNA from the fibroblast cell lines was also analysed by reverse transcription polymerase chain reaction (RT-PCR), and Sanger sequencing of *NFIX* cDNA confirmed that the three remaining MSS fibroblast cell lines (Del1, Del2 and Del3) which carried the previously reported frameshift *NFIX* mutations comprising 3 different deletions spanning introns 5 to 7 (c.819-732_1079-948del (Del1), c.819-471_1079-687del (Del2), c.819-592_1079-808del (Del3)) all resulted in the same change at the cDNA level, namely deletion of exons 6 and 7 (c.819_1079del; p.Ser273Argfs*63). (C) Representative sequence chromatograms showing wild-type *NFIX* cDNA in an unrelated control individual (N1) and mutant *NFIX* cDNA in an affected MSS individual with a large deletion (c.819-592_1079-808del; Del3) that results in the complete loss of exons 6 and 7 (c.819_1079del).

**Supplemental Figure S3. Effect of *NFIX* mutations on the expression of *NFIX* and 2 known downstream target genes in MSS and unrelated control fibroblasts.** qRT-PCR analysis in 5 MSS (Del1, Del2, Del3, InsT and DupG) and 3 unrelated controls (N1, N2 and N3) fibroblast cell lines of (A) *NFIX*, (B) *BBX* and (C) *GFAP* mRNA expression, normalised against *GAPDH* expression and shown as relative expression compared to unrelated controls. (D) Western blot analysis of BBX and GFAP in MSS and unrelated control fibroblasts. GAPDH was used as the loading control. Densitometry analysis of (E) BBX and (F) GFAP expression in the Western blot normalised against GAPDH expression and shown as relative intensity compared to unrelated controls. Data are represented as mean±SEM, n=4, * p<0.05, **p<0.01.

**B**

**A**

**Supplemental Figure S4. Representative scatter plots of RNA sequencing and proteomics data.** (A) RNA sequencing analysis of the *Nfix^Del2/Del2^* MEFs, compared to the mean of *Nfix^+/+^*, *Nfix^+/Del2^*, *Nfix^+/Del24^,* *Nfix^Del24/Del24^*, *Nfix^+/Del140^* and *Nfix^Del140/Del140^* control MEFs, identified 16206 transcripts (grey). Genes that were altered by ≥ 2-fold-change (p<0.05) are shown in blue (downregulated) and green (upregulated). (B) Proteomic analysis of the *Nfix^Del2/Del2^* MEFs, compared to the mean of *Nfix^+/+^*, *Nfix^+/Del2^*, *Nfix^+/Del24^,* *Nfix^Del24/Del24^*, *Nfix^+/Del140^* and *Nfix^Del140/Del140^* control MEFs, identified 4261 proteins (grey). Proteins that were altered by ≥ 2-fold-change (p<0.05) are shown in blue (downregulated) and green (upregulated). The five candidate genes that were either significantly upregulated (*Crabp2*) or downregulated (*Vcam1*, *Kctd12*, *Idi1* and *Eif2s3y*) at the RNA and protein levels are shown in red in both datasets.

**Supplemental Table S1. Fold-change in *Bbx* expression in *Nfix^+/Del2^, Nfix^Del2/Del2^, Nfix^+/Del24^, Nfix^Del24/Del24^, Nfix^+/Del14^* and *Nfix^Del140/Del140^ MEFs* compared with mean of *Nfix^+/+^* MEFs as determined by RNA sequencing analysis.**

| **Compared to *Nfix^+/+^*** | ***Bbx*** | **p value** |
| --- | --- | --- |
| ***Nfix^+/Del2^*** | 1.21 | 0.12 |
| ***Nfix^Del2/Del2^*** | 0.80 | 0.05 |
| ***Nfix^+/Del24^*** | 0.93 | 0.55 |
| ***Nfix^Del24/Del24^*** | 0.86 | 0.21 |
| ***Nfix^+/Del140^*** | 1.06 | 0.62 |
| ***Nfix^Del140/Del140^*** | 0.92 | 0.48 |
|  | | |

**Supplemental Table S2. List of candidate genes in which expression is altered by ≥ 2 fold (p<0.05) in *Nfix^Del2/Del2^* MEFs compared to the mean of *Nfix^+/+^, Nfix^+/Del2^, Nfix^+/Del24^, Nfix^Del24/Del24^, Nfix^+/Del140^* and *Nfix^Del140/Del140^* MEFs from the RNA sequencing and proteomics analyses.**

| **Gene** | **RNA fold-change** | **Protein fold-change** | **p value** |
| --- | --- | --- | --- |
| ***Crabp2*** | 2.59 | 2.83 | 0.012 |
| ***Vcam1*** | -2.23 | -4.89 | 0.014 |
| ***Kctd12*** | -3.53 | -4.77 | 0.049 |
| ***Idi1*** | -5.90 | -2.14 | 0.001 |
| ***Eif2s3y*** | -7305.15 | -5.28 | 0.009 |
|  |  |  |  |

**References**

1.	Priolo M, Schanze D, Tatton-Brown K, Mulder PA, Tenorio J, Kooblall K, et al. Further delineation of Malan syndrome. Human Mutation. 2018;39(9):1226-37.

2.	Zenker M, Bunt J, Schanze I, Schanze D, Piper M, Priolo M, et al. Variants in nuclear factor I genes influence growth and development. American Journal of Medical Genetics Part C-Seminars in Medical Genetics. *181*, 611–626.

3.	Malan V, Rajan D, Thomas S, Shaw AC, Picard HLD, Layet V, et al. Distinct Effects of Allelic NFIX Mutations on Nonsense-Mediated mRNA Decay Engender Either a Sotos-like or a Marshall-Smith Syndrome. American Journal of Human Genetics. 2010;87(2):189-98.

4.	Schanze D, Neubauer D, Cormier-Daire V, Delrue M-A, Dieux-Coeslier A, Hasegawa T, et al. Deletions in the 3 ' Part of the NFIX Gene Including a Recurrent Alu-Mediated Deletion of Exon 6 and 7 Account for Previously Unexplained Cases of Marshall-Smith Syndrome. Human Mutation. 2014;35(9):1092-100.
